# Effect of *Cirsium japonicum* Flower Extract on Skin Aging Induced by Glycation

**DOI:** 10.3390/molecules27072093

**Published:** 2022-03-24

**Authors:** Sohyun Yoon, Minkyung Kim, Seoungwoo Shin, Jieun Woo, Dahee Son, Dehun Ryu, Jiseon Yoo, Deokhoon Park, Eunsun Jung

**Affiliations:** Life Science Institute, BioSpectrum, Inc. A-1805, U-TOWER, 767, Sinsu-ro, Suji-gu, Yongin-si 16827, Gyeonggi-do, Korea; biosw@biospectrum.com (S.Y.); naiad5@naver.com (M.K.); biost@biospectrum.com (S.S.); biotw@biospectrum.com (J.W.); nrz645@naver.com (D.S.); biosc@biospectrum.com (D.R.); biomo@biospectrum.com (J.Y.); pdh@biospectrum.com (D.P.)

**Keywords:** *Cirsium japonicum* flower extract, anti-glycation, skin aging, extracellular matrix, skin elasticity, skin wrinkle

## Abstract

Advanced glycation end products (AGEs) have recently been increasingly discussed as one factor of skin aging. In this study, we investigated the effects of *Cirsium japonicum* flower (CFE) extract on glycation in relation to skin aging and skin elasticity. Moreover, we learned the main active constituent of CFE that has effects against glycation. To demonstrate the effects of CFE on glycation, we carried out an in vitro glycation study, 3-dimensional culture, and clinical study. As a result, CFE inhibited formation of AGEs in both bovine serum albumin (BSA)/glucose glycation system and aldehyde-derived glycation system. Moreover, CFE reduced Nε-(carboxymethyl), lysine (CML), and carbonylated proteins that increased by glycation. Furthermore, CFE broke crosslinks of collagen–AGEs and inhibited the increase of matrix metalloproteinase-1 (*MMP-1*) gene expression by AGEs. In the 3D culture condition, CFE restored the reduction of collagen gel contraction by glycation. Moreover, apigenin was detected as the main active constituent in CFE that has anti-glycation effects. In the clinical study, we confirmed that CFE has effects on skin wrinkles and skin elasticity. Our findings suggest that CFE can be used as a cosmetic or cosmeceutical ingredient for improving skin elasticity and wrinkles. Regulation of AGEs can be an interesting target for anti-aging.

## 1. Introduction

The skin is the largest organ of the human body and an important barrier between the body and the environment. Skin aging has been explained by many theories in endogenous or exogenous ways. Advanced glycation end products (AGEs) that are proteins or lipids that become glycated after exposure to sugars have recently been increasingly discussed as one factor of skin aging.

Glycation is a non-enzymatic reaction between reducing sugars (such as glucose) and proteins, lipids, or nucleic acids. Glycation should be distinguished from glycosylation, which is an enzymatic reaction [1]. Formation of AGEs is a complicated molecular process involving simple and more complex multistep reactions. During the classical Maillard reaction, electrophilic carbonyl groups of glucose or other reactive sugars can react with free amino groups of amino acids, forming a non-stable Schiff base. Further rearrangement leads to formation of a more stable Amadori product. The Schiff base and Amadori product can react irreversibly with amino acid residues of peptides or proteins to form protein adducts or protein crosslinks. Alternatively, they can undergo further oxidation, dehydration, polymerization, and oxidative breakdown reactions to give rise to numerous other AGEs. Oxygen, reactive oxygen species (ROS,) and redox active transition metals can accelerate AGE formation. When an oxidative step is involved, the products are called advanced glycoxidation end products [2].

These oxidation reactions can lead to the formation of more permanent, irreversible chemical modifications and crosslinks of AGEs with protein, such as Nε-(carboxymethyl), lysine (CML), and pentosidine [3]. However, such crosslinks of AGEs can change physical properties of tissues. Some AGEs can form intermolecular crosslinks between collagen molecules and fiber. The effect of these crosslinks on fibers can modify physical properties, leading to increases of stiffness, breaking load, denaturation temperature, and solubility, but there is a decrease in susceptibility to degradative enzymes [4].

People with diabetes have elevated levels of modified proteins arising from glycation and glycoxidation reactions. The level of glycated products on plasma proteins (e.g., albumin) is <3% in a normal healthy population but is increased very dramatically in people with diabetes. Elevated levels of a number of low molecular-mass aldehydes including glyoxal (GO), methylglyoxal (MGO), 3-deoxyglucosone, and glycolaldehyde (GA) have also been detected in people with diabetes. Glycation reactions can occur more rapidly with reactive aldehydes such as GA, GO, and MGO, than with glucose [5]. Particularly, in the case of diabetic patients, the formation of glycation products is highly accelerated and accumulated in skin collagen [6]. Compared to the general population, diabetic patients more commonly experience aged-appearing skin [7]. Furthermore, type 2 diabetic patients show lower levels of a skin elasticity index than healthy subjects. Previous research has shown that the skin elasticity curve of type 2 diabetic patients is steeper, with a downward shift, than that of healthy subjects, although skin elasticity is decreased for all subjects due to aging. They implied that glycation stress is a major factor in the reduction of skin elasticity [8].

The extracellular matrix (ECM) is composed of two main classes of macromolecules: proteoglycans and fibrous proteins. The fibrous ECM proteins are composed of collagens, elastins, fibronetins, and laminins [9]. ECM is closely related to the skin aging, skin health, and elasticity. Molecular modifications such as AGEs can remain unrepaired and irreversible. They can accumulate with age. Moreover, glycation of dermal collagen and elastic fibers contributes to the stiffness and loss of elasticity, forming wrinkles [10].

*Cirsium japonicum* is a perennial plant distributed worldwide. It has been traditionally used in Asia to treat hemorrhages, hypertension, and hepatitis [11]. Previous research reported that *C. japonicum* contains many flavonoids, including apigenin, acacetin, diosmetin, pectolinarin, hispidulin-7-neohesperidoside, 5,7-dihydroxy-6,4′-dimethoxyflavone, linarin, and luteolin with phytochemical studies [12]. Flavonoids in *C. japonicum*, as the major active constituents, perform many bioactivities such as the provision of protective effects against liver injury [13]. In addition, it has been reported that the *C. japonicum* flower possesses potent anti-cancer efficacy by inactivating Akt1 in the PI3K-Akt signaling pathway [14]. In a previous study, we reported that *C. japonicum* flower extract (CFE) promotes melanogenesis in human epidermal melanocyte and improves depigmentation in human hair follicles [15]. However, other possible novel functions of CFE in skin biology, such as aging, wrinkles, and elasticity, were still unknown. Therefore, the objective of this study was to investigate the effects of CFE on glycation by inducing skin aging in vitro, by 3-dimensional (3D) culture, and in vivo through a clinical study. Also, we learned the main active constituent of CFE that has effects against glycation. We tried to explain the anti-aging effect of CFE in terms of glycation and AGEs in this study.

## 2. Results

### 2.1. Effects of Cirsium japonicum Flower Extract on BSA/Glucose Glycation System

**Figure 1:**
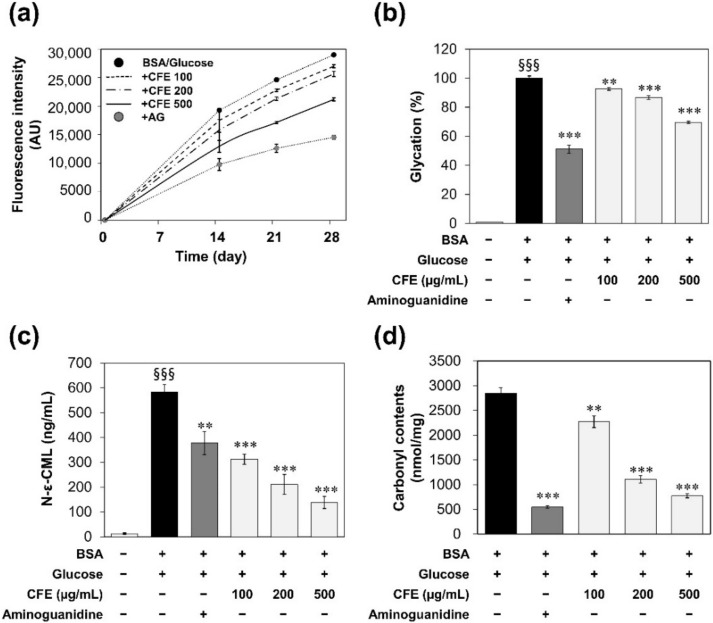
The effects of CFE on BSA/glucose glycation system. (**a**) Fluorescence intensity of AGEs in glucose-modified BSA for 28 days of incubation. These data were represented in the arbitrary unit (AU). (**b**) Percent glycation of glucose-modified BSA at 21 days after incubation. These data were calculated as a percentage (%) of the fluorescence intensity of the sample compared to the control (BSA+/glucose+). (**c**) CML concentration (ng/mL) in glucose-modified BSA at 21 days after incubation. (**d**) Carbonylated protein contents in glucose-modified BSA at 21 days after incubation. These data were represented as the amount of carbonyl per protein in the sample (nmol/mg). Statistical significances were represented as follows: **, *p* < 0.01; ***, *p* < 0.001 compared to the control group; §§§, *p* < 0.001 compared to untreated group.

To investigate the effect of CFE on formation of AGEs, the glycation of bovine serum albumin (BSA) was carried out with glucose for 28 days. Figure 1a shows the formation of AGEs as a fluorescence intensity. The fluorescence intensity of the BSA/glucose control solution was increased gradually over 28 days of incubation. However, fluorescence intensities were decreased significantly after adding 100–500 µg/mL of CFE and 4.5 mM of aminoguanidine (AG). AG was used as a positive control [16]. As shown in Figure 1b, 500 µg/mL of CFE decreased 30.4% of AGEs compared with BSA/glucose control after 21 days of incubation.

CML is one type of AGE. It is a major product of oxidative modification of glycated protein. It has been reported that CML is abundant in vivo where its concentration in collagen tends to be twice as high in diabetic patients as in non-diabetic individuals [17]. Therefore, CML has been suggested as a general marker of oxidative stress and long-term damage to proteins in aging, atherosclerosis, and diabetes [18]. As shown in Figure 1c, CML concentration in BSA/glucose glycation system was also increased after 21 days of incubation. However, CFE inhibited the formation of CML in a dose-dependent manner. CFE at 500 µg/mL reduced the formation of CML by 76.3% compared to the control. This inhibition rate was more than double that of 4.5 mM AG (35.5%).

In addition, protein oxidation was evaluated by measuring the carbonyl group content of the protein (Figure 1d). Glycation generates protein-bound carbonyls, which have been recognized as universal markers of oxidative stress [19]. CFE reduced carbonylated protein in a dose-dependent manner. After 21 days of incubation within the BSA/glucose glycation system, 100, 200, and 500 µg/mL CFE groups reduced protein carbonyl contents to 70.7%, 38.8%, and 27.1%, respectively, compared to the control.

### 2.2. Effects of Cirsium japonicum Flower Extract on Aldehyde-Derived Glycation

**Figure 2:**
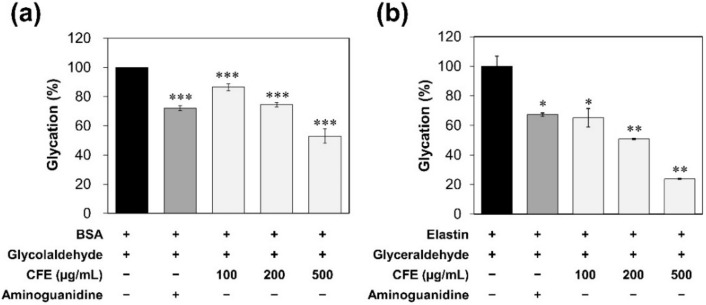
The effects of CFE on aldehyde-derived glycation. (**a**) Fluorescence intensity of AGEs in GA-modified BSA for 5 days of incubation. These data were represented as a percentage (%) of the fluorescence intensity of the sample compared to the control (BSA+/GA+). (**b**) Glycation degree of elastin glycated by glyceraldehyde. Statistical significances were represented as follows: *, *p* < 0.05; **, *p* < 0.01; ***, *p* < 0.001 compared to control group.

The formation of AGEs in vivo is induced by multiple sources and mechanisms involving oxidative and nonoxidative chemical reactions between reducing sugars, Schiff bases, Amadori adducts, and metabolic intermediates such as glyceraldehyde and glycolaldehyde [20]. α-hydroxyaldehydes, such as glyceraldehyde and glycoladehyde have been reported that can induce glycation with rabbit serum albumin (RSA) and form AGE-RSA in close agreement with glucose-modified AGE-RSA [21]. Also, AGE-RSA which was modified by α-hydroxyaldehydes showed very extensive crosslinking that covalently linked adducts of RSA had been formed nonenzymatically. As with glucose-derived glycation, CFE inhibited GA-derived glycation in a dose-dependent manner (Figure 2a). CFE at 100, 200, and 500 µg/mL reduced glycation by 13.5%, 25.6%, and 47.0%, respectively, whereas 1 mM AG inhibited glycation by 17.9%.

Accumulating glycation adducts in ECM can cause physical and structural changes of the skin in relation to aging. Elastin, one of the key ECM components, provides resilience and elasticity to tissues and skin [22]. Previous research has reported that fluorescent AGEs produced by glycation of elastin under the in vitro model can reduce skin elasticity or skin slackening [23]. We observed whether CFE could prevent elastin glycation by inducing glycation of elastin with glyceraldehyde. CFE at 100, 200, and 500 µg/mL reduced the glycation of elastin by 34.9%, 49.2%, and 76.2%, respectively, compared to the control, whereas 1 mM AG caused a 32.7% decrease (Figure 2b). These results demonstrate that CFE can be a potential ingredient with an anti-glycation activity.

### 2.3. Effects of Cirsium japonicum Flower Extract on Glycation of Collagen

**Figure 3:**
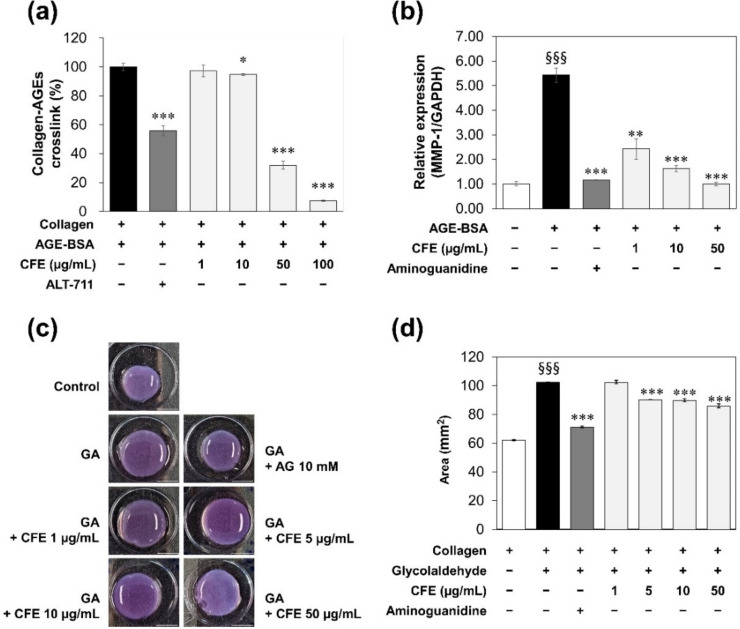
Effects of CFE on glycation of collagen. (**a**) The breaking activity on crosslinks of collagen–AGEs. These data were represented as a percentage (%) of the absorbance of the sample compared to the control (collagen+/AGE+). (**b**) *MMP-1* expression in normal human fibroblast. The level of mRNA transcripts encoding *MMP-1* and *GAPDH* were determined by RT-qPCR after 72 h treatment of AGE-BSA, AG, and CFE. These data were represented in relative expression of *MMP-1* to *GAPDH*. *GAPDH* is a housekeeping gene used as a control. (**c**) The images and (**d**) the areas of 3D collagen lattices after inducing 72 h gel contraction. Statistical significances were represented as follows: *, *p* < 0.05; **, *p* < 0.01; ***, *p* < 0.001 compared to the control group; §§§, *p* < 0.001 compared to the untreated group.

Collagen, the main structural protein in the ECM, can form crosslinks within two specific sites of N-terminal and C-terminal ends. However, AGEs can react with free amino groups on an adjacent protein of collagen to form permanent crosslinks, which can cause the tissue to lose its elasticity and flexibility [24]. To observe the effect of CFE on crosslinks between collagen and AGEs, CFE at 1, 10, 50, and 100 µg/mL were used for treatment after inducing collagen–AGEs crosslinking by GA derived-AGE-BSA. As a result, CFE broke collagen–AGEs crosslinking (Figure 3a). A total of 100 µg/mL of CFE presented a twofold higher breaking effect (92.4%) than 1 mM ATL-711 (44.2%), known as a crosslink breaker for AGEs [25]. CFE also regulated the expression of *MMP-1* known as collagenase-1. After treating normal human fibroblasts with AGE-BSA, the expression of *MMP-1* was increased significantly. However, CFE dose-dependently reduced the increase of *MMP-1* expression (Figure 3b).

Excessive accumulation of AGEs in aged human tissues has been reported [26]. Tissues in different degenerative diseases were observed to stiffen, which might accelerate protein oxidation, altering their structure and threatening their function. ECM stiffness can be changed by increasing the cross-linking through non-enzymatic glycation and accumulation of AGEs. Accumulation of AGE-modified collagen in ECM can impact mechanical properties and cellular interactions known to be important for morphology, structural integrity, and signaling. To evaluate effects of CFE on mechanical properties and cellular interactions of AGE-modified ECM, collagen contraction was observed by culturing fibroblasts within 3D collagen lattices glycated by GA. As a result, 500 µM GA increased the area of a floating collagen lattice compared to the control group by interrupting contraction and making it less deformable. This effect of GA was inhibited significantly by treatment with 10 mM AG, an inhibitor that could prevent the formation of AGEs. Incubation of collagen lattices with concentration of CFE greater than 5 µg/mL reduced the area of the collagen lattice, suggesting that CFE could restore the reduction of collagen contraction by glycation (Figure 3c,d). These results imply that CFE can affect collagen–AGEs crosslinks and *MMP-1* gene expression, which can result in making the collagen lattice contract again so that it is nondeformable by glycation.

### 2.4. Effects of Apigenin and Chlorogenic Acid in Cirsium japonicum Flower Extract on Glycation

**Figure 4:**
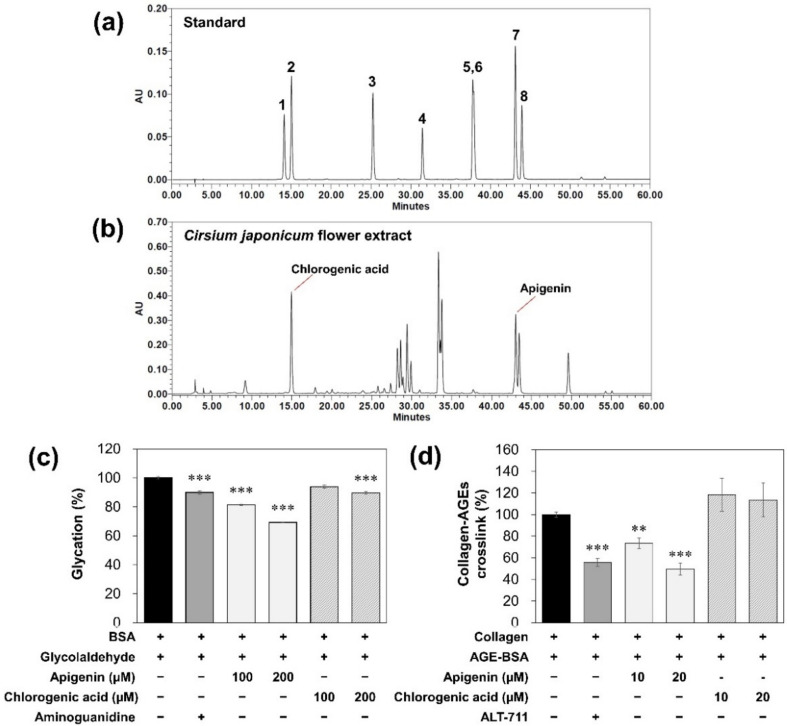
Effects of apigenin and chlorogenic acid in CFE on glycation. (**a**) Analysis of standards composed of flavonoids and phenolic compounds. (1) Scopolin; (2) chlorogenic acid; (3) scopoletin; (4) myricetin; (5) quercetin; (6) luteolin; (7) apigenin; (8) kaempferol. (**b**) Analysis of CFE. (**c**) Fluorescence intensity of AGEs in GA-modified BSA for 5 days of incubation. These data were represented calculated as a percentage (%) of the fluorescence intensity of the sample compared to the control (BSA+/GA+). (**d**) The breaking activity on cross-links of collagen–AGEs. These data were represented calculated as a percentage (%) of the absorbance of the sample compared to the control (collagen+/AGE+). Statistical significances were represented as follows: **, *p* < 0.01; ***, *p* < 0.001 compared to control group.

*Cirsium* species have been known to contain not only various flavonoids including apigenin, luteolin, and kaempferol, but also phenolic compounds such as chlorogenic acid [12,27,28]. To find the active constituents in CFE which affect glycation, we analyzed CFE by using high-performance liquid chromatography (HPLC) and compared it with standards including representative flavonoids and phenolic compounds (scopolin, chlorogenic acid, scopoletin, myricetin, quercetin, luteolin, apigenin and kaempferol). As shown in Figure 4a,b, CFE contains 0.88% (*w/w*) of apigenin and 1.49% (*w/w*) of chlorogenic acid.

Therefore, we examine effects of apigenin and chlorogenic acid on AGEs formation and crosslinks of collagen–AGEs. As a result, apigenin showed a reduction in the formation of AGEs and a breaking of the crosslinks between collagen and AGEs (Figure 4c,d). Totals of 100 µM and 200 µM of apigenin reduced 18.7% and 30.7% of AGEs formation, respectively, whereas chlorogenic acid reduced 10.5% of AGEs formation at 20 µM concentration. Furthermore, 10 and 20 µM of apigenin broke 26.5% and 50.2% of crosslinks between collagen and AGEs. However, chlorogenic acid did not affect the crosslinking of collagen-AGEs. These results suggest that apigenin is one of the main active components in CFE and has effects on anti-glycation and breaking of collagen–AGEs crosslinks.

### 2.5. Anti-Aging Effect of Cirsium japonicum Flower Extract on Human Skin

**Figure 5:**
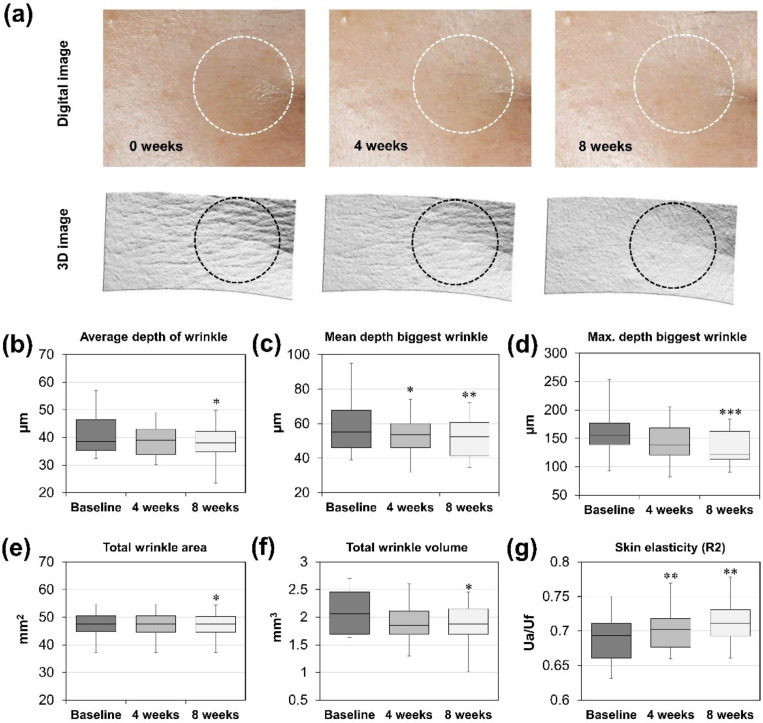
The anti-aging effect of CFE on human skin. (**a**) Skin wrinkles images following 8 consecutive weeks application of CFE. (**b**–**f**) Statistical analysis of skin wrinkles. (**b**) Average depth of wrinkle. (**c**) Mean depth of the biggest wrinkle. (**d**) Maximum depth of the biggest wrinkle. (**e**) Total wrinkle area. (**f**) Total wrinkle volume. (**g**) Statistical analysis of skin elasticity. Statistical significances were represented as follows: *, *p* < 0.05; **, *p* < 0.01; ***, *p* < 0.001 compared to baseline.

To investigate the effect of CFE in vivo, we conducted a human clinical study and observed changes of wrinkles around human eyes. After applying a lotion containing 0.025% CFE for eight weeks, the depth and volume of wrinkles were decreased significantly. As shown in Figure 5a, large and small wrinkles around the eyes were reduced in the 3D image. As a result of observing changes of wrinkle parameters, average wrinkle depths of each subject were gradually decreased over the course of the clinical study (Figure 5b). Mean depths of the biggest wrinkles decreased significantly. In particular, the maximum depth of the biggest wrinkle was reduced by 15.8% compared to the baseline (Figure 5c,d), whereas the total wrinkle area was rarely changed. The total volume of wrinkles was reduced by 8.7% compared to the baseline (Figure 5e,f). Furthermore, skin elasticity increased clearly after 8 weeks of applying CFE (Figure 5g). These results show that CFE can improve wrinkles as well as skin elasticity, suggesting that it can be used as an anti-aging agent.

## 3. Discussion

Skin aging deteriorates many functions of the skin and includes loss of elasticity and formation of wrinkles. Previous research has reported that the reduction of skin elasticity with aging is caused by a reduction of elastic fiber production (i.e., collagen, elastin, and extracellular matrix ingredients such as fibronectin) possibly due to some disorder in fibroblast function [8]. Collagen protein also deteriorates due to oxidation or glycation. In this study, we found that AGEs were formed through glycation of protein induced by glucose or aldehyde. However, CFE effectively prevented not only formation of AGEs, but also CML and carbonylated protein (Figure 1 and Figure 2).

Over time, glycation adducts on a protein chain may covalently bond to a second protein chain, forming permanent crosslinks between protein chains. Although collagen and elastin are strengthened by beneficial crosslinks that do not involve sugar, additional glycation adducts and crosslinks can directly interfere with mechanical properties of structural collagen and elastin fibers, generating pathogenic consequences. Glycation crosslinks can bind to adjacent protein strands, reducing flexibility and elasticity of the tissue [29]. Therefore, breaking glycation crosslinks is an important strategy for maintaining skin elasticity and improving wrinkles. CFE showed a breaking effect on glycation crosslinks between collagen and AGEs (Figure 3a).

In the skin, type I collagen is the most abundant subtype of collagen, followed by small amounts of type III collagen. Fibroblasts located within the dermis mainly synthesize collagen, which imparts strength and elasticity to skin epidermal keratinocytes. Dermal fibroblasts mainly secrete *MMP-1* (interstitial collagenase, or collagenase 1), a collagenase that degrades fibrillar collagens types I and III into specific fragments at a single site within the central triple helix [30]. As shown in Figure 3b, CFE inhibited *MMP-1* expression induced by AGEs, indicating that CFE might be as an intensive cosmetic ingredient for improving skin elasticity.

To confirm this possibility, we cultured fibroblasts within the 3D collagen gel lattice glycated by GA for ECM mimicking (Figure 3c,d). We observed that CFE inhibited the reduction of collagen lattice contraction by GA that increased the area of the collagen lattice. It has been reported that glycation by glucose-6-phosphate decreases collagen lattice contraction but increases tension by forming large well-defined stress fibers without changing the attachment of fibroblasts spreading in the glycated collagen matrix [31]. Crosslinking by glycation can increase the mechanical stability on collagen, which correlates with a reduction in collagen lattice contraction. The stability of collagen fibrils through AGE-derived crosslinks can resist cellular forces exerted by fibroblasts on the collagen matrix. However, CFE could inhibit the reduction of collagen gel contraction by GA to prevent formation of AGEs and break crosslinks between collagen and AGEs.

In order to find the main active constituent showing these anti-glycation effects of CFEs, we analyzed flavonoids and phenolic compounds in CFE and detected apigenin and chlorogenic acid (Figure 4a,b). Apigenin in CFE has also been detected by LC/MS in previous research [32]. In Figure 4c,d, apigenin showed an inhibitory effect on AGE formation and a breaking effect on collagen–AGEs crosslinks. Previous studies have reported that apigenin inhibits the formation of AGEs and AGEs-induced oxidative stress [33], which is consistent with this study. Therefore, these results imply that apigenin in CFE may be the main active constituent for anti-glycation effects.

Effects of CFE on AGEs also showed improvement of wrinkles and skin elasticity in a clinical study (Figure 5). Both the depth and volume of wrinkles around the eyes were decreased after 8 weeks of applying CFE on the skin of 23 female subjects. Skin elasticity in vivo was also improved by applying CFE, consistent with our in vitro experimental results. These results suggest that effects of CFE on AGEs could improve skin elasticity and wrinkles.

## 4. Materials and Methods

### 4.1. Preparation of Cirsium japonicum Flower Extract

*C. japonicum* flower was obtained from Jeju Island, Korea. The raw material was dried in a hot air dryer at 50 °C for 24 h and then pulverized into powder with the particle size ranging from 50 to 200 meshes. Dried *C. japonicum* flower powder (100 g) was extracted with 70% (*v/v*) ethanol at room temperature. After filtration, the solvent was removed with a rotary vacuum evaporator (Heidolph, Schwabach, Germany) to obtain CFE for the cosmetic ingredient. CFE was dissolved in dimethyl sulfoxide (DMSO) only for in vitro laboratory experiment.

### 4.2. In Vitro Bovine Serum Albumin Glycation Assay

#### 4.2.1. Bovine Serum Albumin-Glucose Glycation Assay

The formation of AGEs in BSA was induced by glucose according to a previously published method with slight modifications [34]. Briefly, 10 mg/mL BSA (PanReac AppliChem, Darmstadt, Germany) and 0.5 M d-(+)-glucose (Sigma-Aldrich, Saint Louis, MO, USA) were prepared in 0.1 M phosphate buffer (pH 7.4). CFE (100 to 500 µg/mL) and 4.5 mM aminoguanidine hydrochloride (AG, Sigma-Aldrich, Saint Louis, MO, USA) were incubated with BSA and glucose at 37 °C for 28 days. AG was used as a positive control for inhibiting glycation. Incubated samples were used to detect the formation of AGEs, CML, and carbonylated protein at 14, 21, and 28 days after incubation. Fluorescent AGEs in glucose-modified BSA were detected with an excitation wavelength of 340 nm and an emission wavelength of 430 nm. The percentage of glycation was calculated as the fluorescence intensity of the sample compared to the control (BSA +/Glucose +).

#### 4.2.2. Bovine Serum Albumin-Glycolaldehyde Glycation Assay

GA-modified BSA was prepared by following the method described in a previous study [35] with slight modifications. Briefly, 10 mg/mL BSA, dissolved in 0.1 M phosphate buffer, was incubated with 10 mM GA and 0.02% sodium azide at 37 °C in the presence or absence of CFE (100–500 µg/mL), apigenin (100–200 µM), chlorogenic acid (100–200 µM), or 1 mM AG. After 5 days of incubation, the fluorescence intensity of AGEs was determined with an excitation wavelength of 340 nm and an emission wavelength of 430 nm. Glycation levels are expressed as a percentage of fluorescence compared to the control group (BSA+/GA+).

### 4.3. Enzyme-Linked Immunosorbent Assay

#### 4.3.1. Determination of Nε-(carboxymethyl) Lysine

CML in glucose-modified BSA solution was detected by using an OxiSelect™ Nε-(carboxymethyl) lysine competitive ELISA Kit (Cell Biolabs, INC., San Diego, CA, USA) on day 21 of incubation. Assays were performed according to the protocol of the manufacturer. The content of CML was determined by comparison with a provided predetermined CML-BSA standard.

#### 4.3.2. Detection of Collagen–AGE Crosslinks

The breaking activity on AGE-BSA induced crosslinks to collagen was measured by using a published method [36] with a modification. Briefly, GA-modified AGE-BSA was linked to horseradish peroxidase (HRP) by using the peroxidase labeling kit-NH2 (Dojindo, Tokyo, Japan) according to the technical manual. AGE-BSA labeled with HRP (AGE-HRP) was added to collagen-coated 96-well plates (Advanced Biomatrix Inc., Carlsbad, CA, USA) and incubated at 37 °C for 4 h to form crosslinks between collagen and AGEs. Unattached AGE-HRP was washed with phosphate-buffered saline containing 0.05% Tween 20. Then, CFE (1 to 100 µg/mL), apigenin (10 to 20 µM), chlorogenic acid (10 to 20 µM), or 1 mM ALT-711 (alagebrium chloride, BOC Sciences, Shirley, NY, USA) were used for treatment at 37 °C for 16 h. ALT-711, a novel AGEs crosslinkage breaker, was used as a positive control. TMB solution was added to detect the presence of peroxidase bound to AGE-BSA, which was attached to collagen. The ability of CFE to break collagen–AGE crosslinks was measured by using an Epoch Absorbance Reader (BioTek, Santa Clara, CA, USA) at 450 nm.

### 4.4. Protein Carbonyl Assay

Protein carbonyl content in glucose-modified BSA solution was determined on day 21 of incubation according to a previously reported method [37]. Briefly, 10 mM DNPH dissolved in 2 M HCl was incubated with glycated BSA samples in the dark at room temperature. After 1 h of incubation, cold trichloroacetic acid was added to samples, and protein pellets were obtained by centrifugation at 13,000 pm for 2 min. The pellet was resuspended in 6 M guanidine-HCl and then measured at 370 nm by using a plate reader. The protein carbonyl content of each sample was determined based on the extinction coefficient (ε = 22,000 M^−1^ cm^−1^) for DNPH at 370 nm and calculated as carbonyl to protein (nmol/mg).

### 4.5. Elastin Glycation Assay

The production of glycated elastin was evaluated by using a commercial elastin glycation assay kit (Cosmo Bio Co. Ltd., Tokyo, Japan). Elastin solution was added to a 96-well black plate (Greiner Bio-one, Frickenhausen, Germany). Then 1 mM AG and 100, 200, or 500 µg/mL CFE was added to each well, followed by the addition of 500 mM glyceraldehyde solution. After mixing well, the fluorescence intensity was measured immediately (fluorescent intensity A, Fa) at an excitation wavelength of 370 nm and an emission wavelength of 440 nm by using a fluorescent microplate reader (Infinite F200 PRO, Tecan, Männedorf, Switzerland). The plate was incubated at 37 °C for 5 days and the fluorescence of AGEs found in elastin glycated with glyceraldehyde was measured (fluorescent intensity B, Fb). The glycation degree was calculated as follows:

Glycation (%) = [(Fb − Fa)/(Fb − Fa)control] × 100.

### 4.6. Real-Time Quantitative PCR

Levels of mRNA transcripts encoding *MMP-1* and glyceraldehydes-3-phosphate dehydrogenase (*GAPDH*) genes were determined by RT-qPCR. A normal human fibroblast (primary dermal fibroblast normal; human, neonatal; PCS-201-010) cell line was obtained from ATCC (Manassas, VA, USA). Normal human fibroblast cells (passage 8) were seeded and incubated for 24 h in Dulbecco’s modified Eagle’s Medium (DMEM, Welgene Inc., Gyeongsan-si, Korea) and supplemented with 10% fetal bovine serum (FBS, Welgene Inc., Gyeongsan-si, Korea). Samples diluted in serum-free DMEM were then used to treat cells. The following treatment groups were used: (1) non-treatment as control; (2) 200 µg/mL glycolaldehyde-AGE modified BSA (AGE-BSA, BioVision Inc., Milpitas, CA, USA); (3) 200 µg/mL AGE-BSA with 450 µM AG; and (4) 200 µg/mL AGE with 1, 10, or 50 µg/mL CFE. After 48 h, cells were treated with samples once more and incubated for 24 h.

Total RNA was extracted from cells by using TRIzol reagent (Thermo Fisher Scientific, Carlsbad, CA, USA). RNA concentration and quality analysis were performed by using an Epoch Absorbance Reader (BioTek, Santa Clara, CA, USA). cDNA was synthesized by using amfiRivert a cDNA Synthesis Platinum Master Mix (GenDepot, Katy, TX, USA) according to the manufacturer’s instructions. Primers (Bioneer Corporation, Daejeon, Korea) for amplification of selected *MMP-1* and *GAPDH* are shown in Table 1. *GAPDH*, a housekeeping gene, was used as a control.

The cycling program consisted of denaturation at 60 °C for 1 min, annealing at 25 °C for 5 min, and extension for 30 min, followed by RT inactivation at 85 °C for 1 min. Amplified products were stained by Ampigene qPCR Green Mix Hi-ROX (Enzo Life Sciences, Inc., Farmingdale, NY, USA). RT-qPCR was performed by using a 7500 Real-Time PCR System (Applied Biosystems, Waltham, MA, USA).

### 4.7. Collagen Gel Contraction Assay

A collagen gel contraction assay was performed according to a previously reported method [38] with slight modifications. Briefly, normal human fibroblasts (passage 8, PCS-201-010, ATCC, Manassas, VA, USA) were detached from the culture plate by using trypsin-EDTA (TrypLE Express, Gibco Life Technologies, Grand Island, NY, USA) and centrifuged at 1000× *g* for 3 min. After suspending the cell pellet, cells were counted and a concentration of 4 × 10^5^ cells/mL was prepared by using DMEM. Suspended cells were mixed with collagen gel consisting of 5 mg/mL 3-D culture matrix rat tail collagen I (R&D systems, Minneapolis, MN, USA) and 0.5 N NaOH. Then 500 µL of mixture was immediately transferred to each well of a 24-well plate and incubated at 37 °C for 24 h with humidified 5% CO_2_. After incubation, a gelatinized mixture was gently dissociated from the wall with a pipet tip. Samples were diluted in DMEM containing 1% FBS. The following was prepared: non-treatment as control; 0.5 mM GA; 0.5 mM GA with 10 mM AG; and 0.5 mM GA with 1, 5, 10, or 50 µg/mL CFE. Then 500 µL of each sample was used to treat each appropriate gel and incubated at 37 °C for 72 h. After incubation, photos of the gel were taken and analyzed with ImageJ from National Institutes of Health (Bethesda, MD, USA).

### 4.8. High Performance Liquid Chromatography

The HPLC system used in this study was a Waters 2695 (Milford, MA, USA), equipped with Waters 2996 Photodiode Array (PDA) Detector. The Empower 2 software was used to control the analytical system and perform the data collection and processing.

HPLC-PDA was performed on a Phenomenex Synergi Hydro-RP (4.6 × 250 mm, 4 μm) column reversed-phase column protected by a C18 guard column from Phenomenex, Inc. (Torrance, CA, USA). Ten μL of fifty ug/mL CFE was injected. The signal was monitored at 325 nm.

The elution system used for the HPLC-PDA assay was a binary high-pressure gradient elution system with mobile phase A (0.1% trifluoroacetic acid in H_2_O) and mobile phase B (acetonitrile). The elution gradients are as follows: 10% organic phase B, hold for 5 min; from 10–30% organic phase B in 25 min (linear gradient); from 30–50% organic phase B in 20 min (linear gradient); from 50–100% organic phase B in 10 min (linear gradient), hold for 10 min; then back to the starting condition in 1 min and re-equilibration for 9 min. The flow rate was 1.0 mL per a min. Each analysis required 80 min, including the re-equilibration time.

Standards for analysis were prepared and purchased as follows: Chlorogenic acid (>98%), apigenin (>98%) and myricetin (>98%) were purchased from TCI (Tokyo, Japan). Luteolin (>97%) and scopolin (>98%) were purchased from ChemFaces (Wuhan, China). Scopoletin (>98%), kaempferol (>97%) and quercetin (>98%) were purchased from Sigma-Aldrich (Saint Louis, MO, USA).

### 4.9. Clinical Study

A clinical study on skin anti-aging effects was conducted by Dermapro Ltd. (Seoul, Korea) according to Good Clinical Practice (GCP) and Standard Operating Procedures (SOPs) for 8 weeks. This study obtained ethical approval from the Ethics Committee of Dermapro Ltd., Seoul, Korea (IRB Certification Number: 1-220777-A-N-02-DICN18123). Twenty-three female subjects with wrinkles (average age: 52.52 years) satisfying the inclusion and exclusion criteria participated in this study. The study inclusion and exclusion criteria were shown in Table 2.

These subjects voluntarily decided whether to participate as research subjects and filled out an informed consent form after the investigator fully explained about the purpose and procedures of study. All procedures of this study were in accordance with the Declaration of Helsinki. A lotion containing 0.025% CFE was prepared and applied to the face of subject twice a day appropriately.

Skin wrinkles and elasticity were evaluated before treatment and at four and eight weeks after treatment. Skin wrinkles of crow’s feet were measured with a Primos^®^ Premium (GFMesstechnik GmbH, Teltow, Germany) fitted with a 3D image-analyzing system. Skin wrinkle parameters including average depth of wrinkles, mean depth of the biggest wrinkle, the maximum depth of the biggest wrinkle, the total wrinkle area, and the total wrinkle volume were analyzed by using Primos 5.8 E version software. Skin elasticity of cheeks was analyzed with R2 parameter, an indicator of gross elasticity, by using a Cutometer^®^ MPA580 (Courage + Khazaka electronic GmbH, Köln, Germany) with the principle of suction and elongation.

### 4.10. Statistical Analysis

All statistical analyses were performed with Student’s t-test and analysis of variance (ANOVA) by using Microsoft Excel and SPSS software and expressed as mean ± standard deviation. Statistical significance was considered at *p* < 0.05 and represented as follows: *, *p* < 0.05; **, *p* < 0.01; ***, *p* < 0.001 compared to control group; §§§, *p* < 0.001 compared to untreated group.

## 5. Conclusions

In this study, we observed the effects of CFE on AGEs in vitro, in vivo, and 3D culture condition. CFE showed inhibitory effects on formation of AGEs and breaking effect on crosslinks between collagen and AGEs. Also, apigenin was detected in CFE and showed anti-glycation effects, suggesting that apigenin is one of the main active constituents for effects of CFE on AGEs. We confirmed that the effects of CFE could affect skin elasticity and wrinkles with 3D collagen gel contraction assay with fibroblast and a clinical study applying on the human skin. In conclusion, our findings suggest that CFE can be used as a cosmetic or cosmeceutical ingredient for skin elasticity and wrinkles, and AGEs can be an interesting strategy for anti-aging.

## Figures and Tables

**Table 1:** Specific primers for *MMP-1* and *GAPDH* genes.

Primer	Sequence
*MMP-1*	Forward: 5-ATGAAGCAGCCCAGATGTGGAG-3
	Reverse: 5-TGGTCCACATCTGCTCTTGGCA-3
*GAPDH*	Forward: 5-CATCAAGAAGGTGGTGAAGCAGG-3
	Reverse: 5-AGTGGTCGTTGAGGGCAATGC-3

**Table 2:** The study inclusion and exclusion criteria for clinical study.

Inclusion criteria	(1) Female subjects with wrinkle aged from 50 to 65 (2) Healthy person who does not have acute or chronic physical disease including skin disease (3) Signed and informed consent; the purpose and the protocol of the study were explained to subjects (4) Subjects cooperative and available for follow-up during the study period
Exclusion criteria	(1) Pregnancy or nursing condition or planning to become pregnant (2) Treatment of immune-suppression within one month (3) Participation in a previous study without an appropriate intervening period (three months) between studies (4) Sensitivity or hypersensitivity skin (5) Damaged skin in, or around the test site, which includes sunburn, tattoos, scars or other disfiguration on the test site (6) Use of similar treatment during the previous three months (7) Having an experience on examination site (skin decortications, Botox and other skin treatment) (8) Chronic disease (diabetes, asthma, high blood-pressure) (9) In case of atopic dermatitis (10) Any difficult which may interfere with the aim of the study as the judgment of the investigator

## Data Availability

Not applicable.

## Sample Availability

Sample of *Cirsium japonicum* Flower Extract is available from BioSpectrum, Inc.
